# A Review for the Special Issue on *Paramecium* as a Modern Model Organism

**DOI:** 10.3390/microorganisms11040937

**Published:** 2023-04-03

**Authors:** Judith Van Houten

**Affiliations:** Department of Biology, University of Vermont, Burlington, VT 05405, USA; judith.vanhouten@uvm.edu

**Keywords:** *Paramecium*, ciliate, calcium, calmodulin, symbiont, intron, peripheral protein, mating reactivity

## Abstract

This review provides background and perspective for the articles contributing to the Special Issue of MDPI Micro-organisms on *Paramecium* as a Modern Model Organism. The six articles cover a variety of topics, each taking advantage of an important aspect of *Paramecium* biology: peripheral surface proteins that are developmentally regulated, endosymbiont algae and bacteria, ion channel regulation by calmodulin, regulation of cell mating reactivity and senescence, and the introns that dwell in the large genome. Each article highlights a significant aspect of *Paramecium* and its versatility.

## 1. Introduction 

Reviews about ciliates often cast back to the first documented studies of these cells by the earliest microscopists, who were fascinated with their swimming behavior [1,2]. However, ciliates can be large—*Paramecium* species, for example, ranging from 50–300 μm long—making them easily accessible to a greater number of observers. They thus became endearing to many of us as we observed their acrobatic swimming with low-powered microscopes. This endearment inspired the effective use of ciliates in research, which has grown in popularity and recently been the subject of reviews and two special issues of this journal [3,4,5].

We focus here on the ciliate *Paramecium*, which Denis Lyn called the “white rat” of the Ciliophora due to their usefulness in research. Almost concurrent with a description of a “renaissance” in *P. tetrauralia* research [6] came an exciting discovery of a link between cilia and some human diseases, now known as ciliopathies [7]. As this study will show, ciliates, including *Paramecium,* gained even more momentum as useful model organisms for the study of cilia and provided insights into ciliopathies. *Paramecium* is now the subject of this special journal issue [8], which compiles resources from recent reviews to provide guidance into the use of *Paramecium* in modern research [6,9,10,11,12,13,14,15,16].

Not only are cells of *Paramecium* species large (50–300 μm long), but also, as holotrichous ciliates, they are covered in thousands of cilia. While not multicellular, they are bona fide organisms carrying out functions assigned to multiple different cell types in metazoans (see Preer’s Forward [17] for more details). Within their pond or stream environment, ciliates find food, mate, divide to multiply in numbers, swim by beating their cilia, respond to many kinds of stimuli, control their water balance, release waste and secrete trichocysts [18]. The cells that we feature in this review are in the Phylum Ciliophora, Class Ciliatea, Subclass Euciliata and Order Holotrichida, meaning that we can expect them to be single cells covered all over in cilia. They are of the genus *Paramecium*, which comprises many subspecies [19]. Nyberg and Vivier [20] explain that, over time, the subspecies were divided into two complexes (*aurelia* and *caudatum*), each with their sets of sibling species. *P. tetraurelia*, for example, is in the *aurelia* complex’s subspecies and formerly was *P. aurelia* syngen 4, hence the name “*tetraurelia*”. In addition to sibling species within the two complexes, there are other types, such as *multi-micronucleatum*, *bursaria*, *trichium*, *calkinsi*, *polycaryum*, *woodruffi*, *utrinium* [20]. All are aquatic free swimming and found in fresh water ponds and streams. Several past studies [21] and an article discussed in this special issue [22] contain a select phylogenetic tree. Species diversity of *Paramecium* may be higher than we portray here due to the existence of cryptic species [23]. For example, *P. fokini* n.sp. may be a cryptic species to *P. multimicronucleatum*, which can be distinguished only by molecular methods. However, cryptic species are not among the species described in this special issue.

Species discussed in this special issue are *P. tetraurelia* [24], *P. bursaria* [25], *P. caudatum* [26,27], 6 *Paramecium* species referenced by [28] and even more species in discussed by [22].

**Figure 1:**
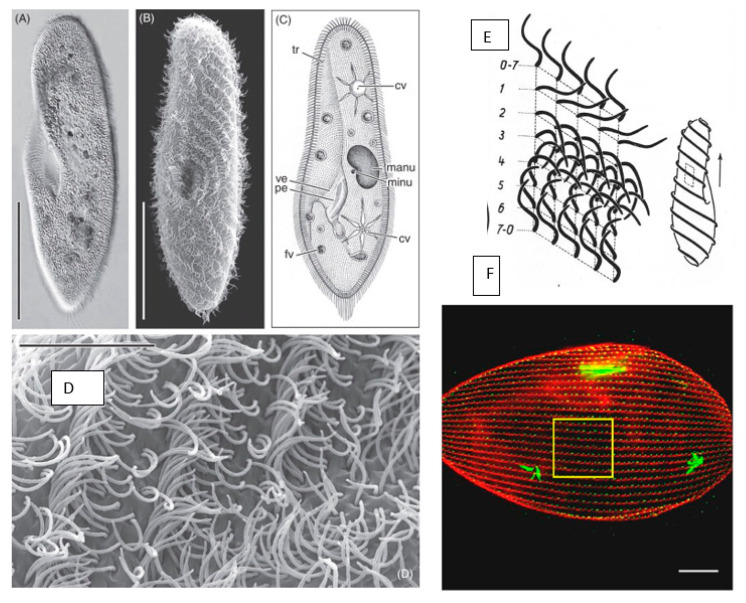
Panel 1 (**A**,**B**): light microscopic appearance of *Paramecium caudatum*. Panel 1 (**C**): drawing of *Paramecium* illustrating light microscopic features: *cv* contractile vacuoles, *fv* food vacuoles, *manu* macronucleus, *mino* micronucleus, *pe* peristome, *tr* trichocysts and *ve* vestibulum. Panel 1 (**D**): higher magnification of the metachronal waves of the cilia. Scale bar in (**A**) and (**B**) 100 μm; (**D**) 10 μm. from Figure 1 in [29] and used with permission. Panel (**E**): form of ciliary stroke and metachrony. Diagrams of instantaneously fixed surface area with five ciliary rows with 0–2 effective, 2–7 recovery stroke. Cell diagram at left shows source of metachronal waves in forward movement. From Grell [32] and used with permission. Panel (**F**): immunofluorescence image of *P. tetraurelia.* Basal bodies green (1D5 antibody) and striated rootlets of the basal bodies red (anti-–SR). Straight rows extend between the anterior and posterior poles. [33]; used with permission.

Hausmann and Allen [29] have documented *Paramecium* with beautiful microscopic images (Figure 1A,B,D). Some of their images and diagrams in Figure 1A–C show *Paramecium* as an asymmetrical cell with two star-shaped contractile vacuoles (cv) for osmotic control. The cilia lining the gullet sweep microscopic cells (usually bacteria, yeast or algae) into the vacuole that forms at the base of the gullet. Structures called trichocysts (tr) that are docked at the cell surface can be forcefully secreted, triggered by the touch of a predator. Figure 1A–C also show two kinds of nuclei: the large polyploid macronucleus (Mac), which is the site of transcription, and the smaller diploid nucleus (Mic), which serves germline transmission. *P. tetraurelia* has two Mics and its large Mac is 800 ploid. As the cells grow and divide, Mics divide by mitosis and move to the poles of the cell. The Mac divides roughly in half, but not by mitosis. Berger, in his review of Görtz’s book titled Cell Cycle, Regulation of Cell Mass and Macronuclear DNA Content [30], refers to the division of the Mac as elongation followed by “amitotic” division and resulting in 5–10% difference in DNA from parental content between the sister Macs. Beisson et al. refer to the Mac division as non-mitotic [31]. Therefore, the new cells receive identical Mics but not necessarily identical Macs. 

In the laboratory, paramecia feed on bacteria and grow into large, dense cultures. To avoid contamination in experiments for protein identification and mass spectrometry, for example, the cells must be washed and treated with antibiotics [9,34]. Alternatively, when just a few cells are needed for crosses or RNA Interference (RNAi), cells can be washed by allowing them to swim upward (negative gravitaxis) several times in tubes filled with sterile buffers, or by purging the bacteria from their food vacuole system by letting them rest in buffer. 

As beautifully described by Jennings more than a century ago, *Paramecium* lives in a rich sensory environment [35]. Dryl described in great detail the swimming behavior and motor responses of *Paramecium* [36]. Paramecia find food, primarily bacteria, by detecting the metabolites and by-products produced by bacterial decomposition in the pond [35,37,38]. In addition, cells move toward the water surface by negative gravitaxis. Upon mechanical stimulation of the anterior caused by bumping into an object or the touch of a predator, the cells back up. Upon stimulation of the posterior, they zoom away from predators [39,40]. *Paramecium* swims against water currents (rheotaxis) and avoids bright light [41] and extremes of temperature. These cells typically swim in helical paths interrupted by abrupt spontaneous changes in direction that Jennings named avoiding reactions. As we will later discuss, the modulation of the frequency of these turns as well as speed modulation form the bases of their biased random walk behavior [42,43,44,45,46]. *Paramecium* is prey for other micro-organisms, such as *Didinium* [47] and *Dileptus*, but *Paramecium* trichocysts appear to provide some protection mechanism against being swallowed by these predators [18,48]. 

## 2. Results

### 2.1. Peripheral Surface Proteins

The most abundant membrane proteins of *Paramecium*, known as surface antigens (SAgs), are peripheral proteins tethered to the cell surface by a glycosylphosphatidyl inositol (GPI) anchor [49]. Ciliary and cell body membranes display these very large proteins that coat the cell and make the cell look fuzzy in transmission electron microscopy [50]. Their functions could include buffering or protection of the cell from environmental insults. Similar proteins in parasitic protozoa are part of strategies to elude host defenses by trypanosomes or *Plasmodium* by expressing a large number of antigens or switching antigens to avoid the host’s antibodies [51]. In *Paramecium*, one strategy of interest, similar to those of antigen switching in parasitic protozoans, is programmed antigenic variation as you will see below.

*Paramecium* surface GPI-anchored proteins are primarily large SAgs, but also include smaller proteins. Among this latter group is a chemoreceptor [52], the evidence for which is both indirect and direct. Indirect evidence has come from antisera: antibodies against *Paramecium* surface antigens specifically block chemoresponse to folate. Presumably, the antisera raised against the GPI-anchored proteins included antibodies against this smaller protein as well. Secondly, antibodies against the mammalian GPI-anchored folate receptor blocks chemoresponse to folate [53]. More recently, RNAi reduction in the first enzyme in the GPI anchor synthesis, PIG-A, has selectively reduced chemoresponse to folate and glutamate [54]. A physical protein that binds folate and acts as a chemoreceptor for the attractant folate has been identified among the GPI-anchored proteins of the cilia and cell surface [52,53,55]. While the folate receptor clearly is part of a signal transduction pathway governing a chemoresponse, the mechanistic details for signaling by these peripheral proteins still need to be determined. The study by [28] discusses further receptor possibilities. 

These GPI-anchored proteins, especially surface antigen (SAg), have come to be known as immobilization antigens because antibodies against them cross-link and prevent the cell from moving [56]. These antigens also relatively quickly change across the entire cell surface and in a particular order after environmental perturbations of temperature or pH; this process is known as programmed antigenic variation [57]. Since there is mutually exclusive expression of these SAg genes in the multigene family, these programmed changes must involve controls at the genome level. Only a few of the genes for surface antigens in *P. tetraurelia* and *P. primaurelia* had until now been studied, making it difficult to understand how SAg genes are transcribed and how the feat of programmed changes in the antigens is accomplished. 

Additionally, the release of the SAgs from the surface for replacement by a new group of proteins seems to be accomplished by Phospholipase C isoforms that are selective for GPI protein cleavage and do not cleave all GPI-anchored proteins at once, which would lead to non-specific shedding of all the SAgs [58]. The SAgs complicate the membrane biochemistry protocols of ciliary and cell body membranes because SAGs are so numerous and the target proteins of interest are much less abundant. Therefore, it is imperative to separate the SAgs from channel proteins or transmembrane receptor proteins of cilia, as shown in [15]. A fortuitous aspect of SAgs comes to the rescue: they are easily cleaved from their anchors. During the membrane isolation process, treatment of the preparation with detergents or exogenous Phospholipase C releases the GPI-anchored proteins into the aqueous phase; they can then be separated from membranes by differential centrifugation [15,34]. 

Pirritano et al. in this special issue [28] present a deep dive into the SAg genes in the genomes of six *Paramecium* species. Their data provide insights into subfamilies, the evolution of these genes and the consensus motifs across species. The motifs that they identified hint at potential functions for these interesting proteins.

### 2.2. Endosymbionts 

Some *Paramecium* species harbor algae or bacteria as endosymbionts. Two of our special issue articles address endosymbiosis.

*P. bursaria* cells become a beautiful green when they are inhabited by the algae *Chlorella*. These *P. bursaria* can be studied with or without the *Chlorella,* setting up an interesting comparison and control. Iwatsuki and Naitoh [41] showed that *P. bursaria* are intrinsically photosensitive, even without symbionts like *Chorella* that are photosynthetic. They point out that *Chlorella* symbionts alter the intrinsic responses of *P. bursaria* to changes in light; Without *Chlorella, P. bursaria* react to the step up in light with an avoiding reaction (AR) and transient depolarization, which help to keep the cells in the shade and out of harmful radiation. *P. bursaria* with *Chorella* show an AR upon step down to lower light, often sending them back to the lighted area where photosynthesis is supported. This behavior is a form of klinokinesis and results in accumulation of cells in the shade, as explained in more detail below. 

Likewise, Matsuoka and Nakaoka [59] observed that *P. bursaria* accumulated in lighted regions; however, in their studies both *Chorella*-containing and C*horella*-free ciliates responded to a step up in light with a steady depolarization. This steady depolarization translated into a slowing of cell swimming speed that would keep ciliates in the area of light. This slowing resulting in accumulation of a population is called orthokinesis and is discussed below. The cells also show an AR upon entering shade that tends to send them back into the lighted areas, thus trapping them in areas of light.

As discussed by Sommaruga and Sonntag [60], these previous studies are difficult to compare because of different cell preparations and protocols for adaptation. What is clear is that *P. bursaria* with *Chlorella* execute behaviors that are familiar to those who analyze *Paramecium* swimming behavior and the physiological correlates associated with electrophysiology. We will further discuss this aspect below under the section on swimming behavior. 

In this special edition, Takahashi [61] takes advantage of what is known about the algae of *P. bursaria* to analyze the effects of environmental stress upon the host and its endosymbionts. One goal was to provide a monitoring method for endosymbiotic systems beyond *P. bursaria*. Temperature rise and toxins, such as paraquat cause corals, lose their algal symbionts and bleach. The system being developed by Takahashi could help to monitor and anticipate the collapse of endosymbiotic systems under stress.

The topic of endosymbiosis in *Paramecium* also has a long history in the study of bacteria such as the ones responsible for the Killer phenotype, in which infected cells kill non-infected ones and become immune to the effects of the Killer bacteria [62,63]. The source of the factor has been identified as the obligate symbiont bacterium *Caedibacter taeniospiralis* [64] and identification of the toxin(s) among secreted candidates is underway. 

Fujishima et al. describe in this special journal issue [27] a different symbiotic system in which a bacterium specifically infects the macronucleus of *P. caudatum. Holospora obtusa* is a gram-negative bacterium that endows its host with heat-shock and high salt tolerance by modifying host cell transcription [65] (read references 1–6 in Fujishima et al. in this special issue for information on *Holospora* species [27]). To investigate how Mac transcription is hijacked by this bacterial symbiont, Fujishima et al. focused on the macronucleus and a 63 kDa protein, showing that it binds to *P. caudatum* DNA.

### 2.3. Mating

A characteristic of ciliates is the presence of two kinds of nuclei: one is diploid and has the expected 2n number of chromosomes for germline transmission of the genome, while the other is highly polyploid (800 ploid in *Paramecium tetraurelia*) for gene expression. In *P. tetraurelia*, there are two diploid micronuclei (Mic) and one polyploid macronucleus (Mac); in *P. caudatum*, there is one Mic tucked next to the large polypoid Mac (reviewed in [6]). 

During the life cycle of *P. tetraurelia*, during the vegetative cell division cycle the cell duplicates its micronuclei by mitosis and elongates its macronucleus. The duplicated micronuclei move to the poles of the cell—two to each pole—to prepare for two new cells that will form from cytokinesis. The macronucleus also divides, though not by mitosis; the products are, therefore, not identical. DNA synthesis in *Paramecium*’s nuclei does not neatly follow rules of other eukaryotes. For example, Mac and Mic DNA replication follow different time regimens in the cell cycle for vegetative cell division and reproduction [30]. Nanney refers to the timing of Mac and Mic DNA synthesis as uncoupled from each other [66]. After cytokinesis there are two cells each, with two micronuclei and one macronucleus (read [31,66] for a review of this subject). 

Other important features of the *P. tetraurelia* are the two sexual processes of mating and autogamy. Starvation of the *P. tetraurelia* cells (and others only of the *P. aurelia* complex) results in a nuclear reorganization; this can progress to mating of complementary cells or, if no complementary cells are available, to autogamy. During mating, starved complementary mating type cells will fuse over part of their surface and exchange haploid nuclei; these nuclei are the products of the multistep process by which the diploid Mic divides by meiosis twice, forming four products of which three disintegrate. The last haploid nucleus divides again, providing one nucleus to donate to the conjugant mate and one to retain. After the cells have exchanged the haploid nuclei, within each cell, the nuclei fuse to form a diploid nucleus. These further divide by mitoses, ultimately providing the two that become micronuclei for the cell and two that will contribute to the process of forming a new Mac. The old Mac disintegrates and a new Mac is formed with directions from the new Mic. In cells entering into conjugation and sexual reproduction, the Mic initiates DNA replication and meiosis late in the cell cycle while the cells are in G2 [30]. Fujishima describes the preparation of Mics for entry into meiosis [67]. At 2–2.5 h after mixing, during the Mic pre-meiotic S phase, the mating reactive cells commit to the degradation of the Mac. The DNA synthesis for the new Mac development (or duplication for adjusting Mac DNA content after vegetative or sexual cycle) is not coupled in time to the Mic DNA S phases (read Section 2.4 for a discussion of Mac development).

In the case of autogamy, the species of *Paramecium* that go through this process become homozygous at all loci in one manipulation in the laboratory (read the excellent diagrams in [6] and [66] that visualize both the nuclear steps in crosses and autogamy and how autogamy makes a second set of crosses to produce an F2 unnecessary). A downside of autogamy is that it fixes in place any mutations that creep into stocks and are selected for if they provide an advantage to the cells in culture [68]. Autogamy starts in the starving vegetative cell with the macronucleus breaking down and fragmenting, while the micronuclei divide by meiosis to produce haploid nuclei followed by one more mitotic division. All but one of these haploid micronuclei disintegrate; the remaining one divides and fuses to form a diploid micronucleus and, after another division, divides again to produce four diploid nuclei. The two at the posterior of the cell differentiate into macronuclei; the ones at the anterior pole remain micronuclei. During the following cytokinesis, these nuclei distribute to the two daughter cells, with each receiving two identical micronuclei and one macronucleus. Fragments of the old macronucleus continue to disintegrate. The important take away is that these new cells are now homozygous at all loci [11]. 

Following mating, the cells are “immature” and unable to mate again for a species-specific time period [69]. In *P. caudatum*, this immature period lasts for about 50 fissions. Using the important technique of microinjection, Haga and Hiwatashi [70] identified a cytoplasmic factor that inhibits mating by mature cells. From the cytoplasm used to inhibit mating, Haga and Hiwatashi isolated a small protein they called immaturin. *P. caudatum* cells age and, when they reach senescence, are unable to mate [70]. Immaturin also has the property of reversing senility in aging cells. This protein is the topic of the article in this special issue written by Haga et al. [26] Their research takes the study of immaturity to the genomic level (for more information about *P. tetraurelia* that has the capacity to undergo autogamy as well as mating, see the recent article by Haga [71]). This article also describes in detail the method and discoveries in *Paramecium* biology using microinjection, including identification of calmodulin as a critical component of ion channels (see below). See [12] for more protocols for microinjection.

### 2.4. Intronization

The *Paramecium* genome is physically different in each of its two forms of nuclei (read [6,72] for excellent overviews, as well as Berger’s review for the timing of DNA replication during the cell cycle [30]). The germline micronucleus of *P. tetraurelia* is diploid with about 50–60 small chromosomes. After each sexual cycle (mating or autogamy), a new macronucleus develops from the micronucleus by amplification of the micronucleus to about 800 ploid. However, not all the sequences of the micronucleus are found in the macronucleus. Instead, many are internally eliminated sequences (IESs) that have been precisely excised from the micronuclear DNA or imprecisely excised to create the Mac. The processes involved in these conversions of micronuclear into macronuclear genomes have been extensively documented. The precise IES removal involves transposases and comparisons of sequences by non-coding RNAs [72]. PiggyMac is a transposase that was acquired, domesticated and now utilized in the precise excision of IES in Mac development [73]. Five additional PiggyMac like proteins interact and participate in the IES excision genome-wide [74]. Imprecise elimination of the germline sequences from the new macronucleus seems to involve the fragmentation of the micronuclear genome [75] to create 200 compact chromosomes of about 50 kb to 1 Mb and characterized by little spacing between genes and coding regions with short introns. The estimated 30,000 genes of *Paramecium* [72,76] are closely packed into the macronuclear genome. This rather high number of genes is due to three genome-wide duplications [77]. 

The introns within the gene exons are very small [78]; however, they should be precisely spliced from mRNAs. Ryll et al. [24] in their contributed article ask us to consider introns, which in some systems are retained in mature mRNA 50% of the time. They use *Paramecium tetraurelia* as a model, which they examine on a genome-wide scale as a snapshot of ongoing evolutionary processes, such as exon-to-intron conversion (and vice versa). The authors remind us that there can be variations in splicing that are important in processes such as the development of immune cells; some intron sequences are retained in exons and serve as opportunities for evolutionary change. They also suggest how introns are gained and eventually expressed, beginning with a splicing error. Other authors would point out that an intron located between the two exons can be either excised or retained; if retained, this intron could be a by-product of alternative splicing. Clearly Ryll et al. challenge our ideas of RNA splicing, introns and exons.

### 2.5. Calcium, Calmodulin and Cilia in Swimming Behavior

#### 2.5.1. We Should Not Leave *Paramecium* without Discussing Their Contribution to Our Knowledge of Cilia and the Spectacular Genetic Dissection of the Roles of Calmodulin

Because cilia structure and function are highly conserved, it is possible to use many different organisms to study the fundamentals of cilia and even the diseases (ciliopathies) arising from their malfunction [79]. While it is expected that many studies of cilia employ mammalian tissues (e.g., kidney cells or lung epithelia), many informative studies also come from a variety of other organisms, such as zebra fish, the algae *Chlamydomonas reinhardtii*, the nematode worm *Caenorhabditis elegans,* trypanosomes and ciliates, such as *Tetrahymena* and *Paramecium*, among many others. *Paramecium* studies benefit from the large number of cilia that can be harvested for biochemical studies and proteomics, compared to the single cilium per cell of the tissues with primary cilia. There are other important research approaches, such as amenability to electrophysiology and generation of mutants, for which *Paramecium* is advantageous, as we explain below.

Paramecia are propelled by thousands of motile cilia that are long (10 µm), thin, membrane-covered organelles protruding from the cell surface. The many cilia beat in metachronal waves (Figure 1D), as well as being physically entrained and not coordinated by any physical or electrical connections [29,80]. The basal bodies from which cilia arise are spaced in a regimented pattern of longitudinal rows from pole to pole across the cell surface (Figure 1F), which is critical to maintain the spacing needed for cilia to beat in metachronal waves. We also noted that the scanning electron microscope images of the waves (Figure 1B,D) capture the cilia in stages of the ciliary beat over time. During forward swimming, the cilia beat with their power stroke toward the posterior of the cell, driving the cell ahead. The return stroke is slower and repositions the cilium for the next power stroke. In Figure 1B,E, the curled cilia are most abundant since the cilia spend the most time in this slow recovery stage of the beat. We can compare the cilia in Figure 1D with the diagram rendered by Parducz in Figure 1E [32,81] to see the stages of the ciliary beat within the metachronal waves. They are also illustrated by Satir [82]. 

The cells also are able to turn and swim backward. In general, the turn consists of a short backward movement, twirling in place and renewed forward swimming in a new direction. This is the basis of the avoiding reaction described by Jennings [35] and mentioned above in the behavior of *P. bursaria* with *Chlorella* as they leave a lighted area. In the change between backward and forward movement, the sweep of the cilium toward the anterior becomes the strong power stroke, and the stroke toward the posterior becomes the lazier return stroke. The cells pivot in place with the cilia straight out as they transition between power stroke patterns. Machemer thoroughly reviews cilia movements during beating [83]. The molecular and physiological bases of these ciliary motions are discussed below.

The cilia that cover *Paramecium* are remarkably similar to cilia across many phyla [10,11,84]. As with other cilia, they grow from base to tip by transport of proteins from a basal body (BB), which docks at the cell membrane to the tip. The BB is characterized by triplets of microtubules and a transition zone between the axoneme and the BB for sorting, through which proteins enter and leave the cilium [85,86] (read the review by Tassin and workers [87] and more recently [10,88] for more details). The cytoskeleton of the cilium is the axoneme, with nine doublet microtubules and two singlet microtubules in the center. The microtubules slide relative to one another, allowing the cilium to bend and beat [89]. Special motor dynein Mg-ATPases interact with the microtubule doublets to move the cilia in their graceful arcs [89]. *Paramecium* cilia beat at about 10–20 Hz. 

#### 2.5.2. Neuronal Properties of Cilia

Physiologists from Japan, Europe and the United States developed the insight that *Paramecium* swimming behavior and the ciliary beat underlying it are under bioelectric control [83,90,91,92,93]. They went on to characterize channels of the cilia that are the fundamental bases for this bioelectric control (reviewed in [94,95,96]). Inevitably, the cells were given the nickname of “little swimming neuron” [97]. 

**Figure 2:**
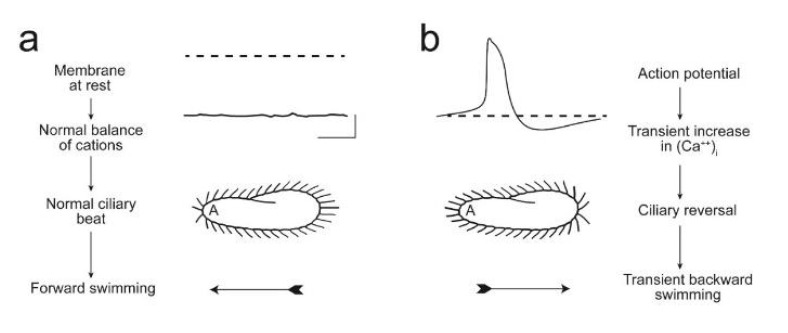
(**a**) These images illustrate that the resting membrane potential of *Paramecium* is negative (about −25 to −40 mV); the corresponding ciliary beat is toward the posterior of the cell and the cell swims forward. (**b**) In depolarizing solutions, such as high K^+^ or Ba^2+^, the cell’s plasma membrane depolarizes and reaches threshold for the action potential. During the action potential, Ca^2+^ enters the cilia through voltage-gated channels; the high levels of Ca^2+^ change the power stroke of the cilia, which now beat most strongly toward the anterior and move the cell backward. The action potential is quickly terminated and the Ca^2+^ removed from or sequestered in the cilia, allowing ciliary beat and swimming to return to normal. From [94] Figure 1; used with permission.

Figure 2 summarizes the understanding of the physiology underlying *Paramecium* forward and backward ciliary beating and the AR. Forward swimming, associated with a power stroke toward the posterior, is associated with the resting membrane potential (Figure 2a). A turn in the swimming path requires a short change in the power stroke causing an AR (Figure 2b). This change is initiated by a depolarization large enough to open the Ca^2+^ channels of the cilia, transiently allowing the Ca^2+^ to reach the axoneme. The power stroke changes transiently toward the anterior and the cell swims backward, usually for a short time. Since the cell is asymmetrical, the new direction of swimming after an AR is usually in a new random direction.

During the AR, the return of the membrane potential to rest is accomplished by two K^+^ conductances: a rapid voltage activated K^+^ conductance (I_(KV)_) and a slower Ca^2+^ activated K^+^ conductance (I_(KCa)_). The depolarization phase of the action potential activates the fast voltage dependent ciliary K channel (K_V_) and the Ca^2+^ that enters the cilium through the Ca_V_ channels during the action potential activates the slower responding calcium-dependent ciliary K channel (K_Ca_) [98,99]. These two types of K channels, similar to the special ciliary Ca^2+^ channels [100], appear to be concentrated in the cilia and reduced or absent from the soma [91,101]. The Ca^2+^ feed-back to inactivate the Ca_V_ channel also contributes to the end of the action potential [101].

Even though the membrane potential returns to rest, cells will continue to swim backward as long as Ca^2+^ remains high in the cilium. The removal or sequestration of ciliary Ca^2+^ is a matter of discussion, further discussion on the role of calmodulin-regulated Ca^2+^ pumps in this process [10,95,102].

The speed of swimming is also under bioelectric control. It depends upon the resting membrane potential, which, in turn, controls ciliary beat frequency [91,103,104]. Small hyperpolarizing stimuli increase beat frequency and swimming speed; small depolarizing stimuli do the opposite.

These basic swimming behaviors (speed and turning) underly more complex swimming feats of populations of cells. For example, paramecia swim to accumulate in or disperse from chemicals in their environment. They do not orient and swim directly toward or away from the source of chemicals by a chemotaxis. Instead, they employ more indirect methods of chemokinesis. Paramecia move in their environment in a random walk, a combination of forward swimming runs and turns due to ARs. If they enter an area with attractant molecules, they move up gradients of attractants by biasing this random walk. They accumulate in attractants by suppressing turns from ARs and increasing speed as they make their way up gradients [45]. In this complex behavior, they employ klinokinesis (turning) and orthokinesis (speed) modulation. Their behavior as they encounter repellents is the converse. If they start to go up a gradient of repellent (or begin to go down a gradient of attractant), they immediately start to turn and swim more slowly and, eventually, move out of the repellent or back into the attractant. 

The fast smooth swimming with few turns in attractants results from hyperpolarization, which increases ciliary beating frequency and suppresses action potentials and turns. In repellents, cells depolarize, which increases the frequency of action potentials and turns and decreases ciliary beating frequency, slowing the swimming speed [105] (read the [38,45] for more details).

(Read Fraenkel and Gunn for an extensive description of behavior of micro-organisms including orthokinesis and klinokinesis [106]; read Manson for a description of biased random walks in bacterial chemoaccumulation [107]; read [38,42,44,108] for more information on *Paramecium* chemoresponse).

Returning briefly to the behavior of the *P. bursaria* with and without *Chlorella* endosymbionts, we see that the cells’ reaction of an AR and an action potential, which is to a step up in light and transient depolarization, keeps these cells in the shade by a klino-kinesis mechanism [41]. Likewise, when cells with *Chlorella* experience a step down to lower light, they have an AR that can send them back into the lighted area.

Matsuoka and Nakaoka [59] observed accumulation of *P. bursaria* (with and without *Chlorella*) in lighted regions by associated with a steady depolarization. Such a depolarization would slow down the cells and keep them, perhaps trapped, in the areas of light by orthokinesis. Upon entering shade, the subsequent AR would reinforce their accumulation in light by a klinokinesis. These behaviors and underlying membrane potential changes are very much like those of *P. tetraurelia* in attractants and repellents.

#### 2.5.3. Calmodulin, Channels and Swimming Behavior

In this special issue, Villalobo et al. [22] provide an extensive review of *Paramecium* calmodulin, including the *Paramecium* behavior described below.

Another advantage of *Paramecium* that was very successfully exploited for the analysis of swimming behavior is the generation of mutants. Kung named one group of mutants Pawn because, like the chess piece, they could not move backward (reviewed in [109]). These mutants were found to lack the Ca_V_ conductance and, more recently, to lack the protein that is the cilia-specific voltage gated Ca channel in their cilia [110].

Other behavioral mutants showed prolonged backward swimming upon depolarization because they lacked the conductance from the K_Ca_ channel to quickly repolarize the cell. Others showed hypoactive response to stimuli that should depolarize, such as Na solutions. Their failure to swim backward stemmed from the failure of Ca to activate Na_Ca_ channels. Genetic analysis showed that these two apparently opposite phenotypes were due to different mutations in the same gene, while microinjection studies showed that the gene product was calmodulin [35,71]. Analysis of a large number of alleles showed that mutations with changes in the C terminal lobe were associated with the hyperactive phenotype and the changes in the N terminal lobe resulted in the hypoactive phenotype. The Kung lab had, remarkably, accomplished a genetic dissection of a molecule that is crucial in the control of ion channels.

We advise readers to refer to the reviews by [12,22,92,104] for discussions of the conductances, channels and receptors for chemoreception in *P. tetraurelia*.

## 3. Conclusions

This collection of articles gives some important insights into the use of *Paramecium* species in research:

- Regulation of gene exclusion and developmentally regulated sets of genes, such as for the surface antigens;
- Harboring eukaryotic and prokaryotic symbionts, which can be evaluated for their impact on their host’s physiology, gene expression and behavior;
- The most conspicuous feature of *Paramecium* is its lively swimming powered by cilia, which, in turn, are controlled by ion channels. Calmodulin is an important player in the Ca^2+^ dependence of many of these channels and other Ca^2+^-dependent cellular processes;
- *Paramecium* cells mate and exchange nuclei, and they also age and senesce. The timing and control of these processes are becoming clear;
- Lastly, the very large genomes and high ploidy provide a great source of material for examination of genome structures and development of introns.

The above studies depend upon some aspects of *Paramecium* that make it a handy modern model organism: large cultures for biochemistry and proteomics; genetic and molecular manipulation (e.g., RNAi); availability of large high ploidy genomes for analysis of genome rearrangements; access to ion channels in cilia and cell body membranes using electrophysiology; visualization of intracellular and surface structures by scanning and transmission of electron microscopy and cryo-tomography; expression of fluorescent-tagged proteins using microinjection and immunofluorescence microscopy techniques to follow location and trafficking; and forward mutant generation followed by whole genome sequencing to identify genes. 

There is a rich assortment of resources available for research on *Paramecium*, such as ParameciumDB, which is a community resource that integrates the *Paramecium tetraurelia* genome sequence with genetic data [2,3,5,6,7].

## Data Availability

This is a review. Data are available from the primary sources.
